# Feeding and fasting controls liver expression of a regulator of G protein signaling (Rgs16) in periportal hepatocytes

**DOI:** 10.1186/1476-5926-5-8

**Published:** 2006-11-23

**Authors:** Jie Huang, Victor Pashkov, Deborah M Kurrasch, Kan Yu, Stephen J Gold, Thomas M Wilkie

**Affiliations:** 1 Department of Pharmacology, UT Southwestern Medical Center, 6001 Forest Park Dr., Dallas TX 75390-9041, USA; 2 Department of Physiology, University of California, San Francisco 94143-2611, USA; 3 Department of Neurology, University of Mississippi Medical Center, Jackson MS 39216, USA; 4 Department of Psychiatry, UT Southwestern Medical Center, 6001 Forest Park Dr., Dallas TX 75390-9070, USA; 5 Department of Pharmacology and Neuroscience, University of North Texas Health Science Center, 3500 Camp Bowie Blvd., Ft. Worth TX 76107, USA

## Abstract

**Background:**

Heterotrimeric G protein signaling in liver helps maintain carbohydrate and lipid homeostasis. G protein signaling is activated by binding of extracellular ligands to G protein coupled receptors and inhibited inside cells by regulators of G protein signaling (RGS) proteins. RGS proteins are GTPase activating proteins, and thereby regulate Gi and/or Gq class G proteins. RGS gene expression can be induced by the ligands they feedback regulate, and RGS gene expression can be used to mark tissues and cell-types when and where Gi/q signaling occurs. We characterized the expression of mouse RGS genes in liver during fasting and refeeding to identify novel signaling pathways controlling changes in liver metabolism.

**Results:**

Rgs16 is the only RGS gene that is diurnally regulated in liver of *ad libitum *fed mice. Rgs16 transcription, mRNA and protein are up regulated during fasting and rapidly down regulated after refeeding. Rgs16 is expressed in periportal hepatocytes, the oxygen-rich zone of the liver where lipolysis and gluconeogenesis predominates. Restricting feeding to 4 hr of the light phase entrained Rgs16 expression in liver but did not affect circadian regulation of Rgs16 expression in the suprachiasmatic nuclei (SCN).

**Conclusion:**

Rgs16 is one of a subset of genes that is circadian regulated both in SCN and liver. Rgs16 mRNA expression in liver responds rapidly to changes in feeding schedule, coincident with key transcription factors controlling the circadian clock. Rgs16 expression can be used as a marker to identify and investigate novel G-protein mediated metabolic and circadian pathways, in specific zones within the liver.

## Background

Body weight homeostasis is maintained through complex communications between the brain and peripheral organs [1-8]. Declining body weight during fasting promotes increased food intake and decreased energy expenditure. By contrast, weight gain following several large meals is compensated by decreased food intake and increased energy expenditure. Many of the orexigenic and anorexigenic signals providing dynamic control of energy and body weight homeostasis are conveyed by G protein coupled receptors (GPCRs) in the brain and periphery [5,9,10]. Here, we investigate a novel approach to understand how G protein signaling in liver regulates metabolic activity to maintain body weight and energy balance.

The activity cycle of heterotrimeric G proteins revolves around receptor-catalyzed guanine nucleotide exchange and GTP hydrolysis on the Gα subunit. In the inactive state, Gα^GDP ^forms a heterotrimeric complex with Gβγ. Hormone binding to GPCRs activates intracellular signaling by catalyzing guanine nucleotide exchange on the Gα subunit [11]. Active Gα^GTP ^and Gβγ subunits dissociate to regulate effector proteins and the subsequent production of second messengers that provoke cellular responses to physiologic stimuli. GTP hydrolysis on Gα restores the inactive heterotrimeric complex of Gα^GDP^βγ. Regulators of G protein signaling (RGS) proteins are GTPase activating proteins (GAPs) for Gi and Gq class α subunits [12-15], and are distantly related to rgRGS proteins that accelerate GTP hydrolysis on G12 class α subunits [16,17]. RGS proteins regulate the specificity, intensity and duration of Gi and Gq signaling [18]. RGS proteins of the R4 family, such as Rgs16, are feedback inhibitors that can terminate signaling by uncoupling hormone binding from effector protein activation [19-21].

A useful characteristic of RGS gene expression is that it can be induced by GPCR agonists and second messengers [22,23]. A paradigm for feedback regulation of G protein signaling by RGS proteins was established by analysis of the yeast mating response [24]. Mating pheromones are GPCR ligands that stimulate cell cycle arrest in haploid cells. The G protein alpha subunit (GPA-1) releases Gβγ to stimulate a MAP kinase cascade resulting in the transcriptional activation of the mating response pathway [25-27]. Interestingly, transcription of the yeast RGS gene *Sst-2 *is also induced by mating pheromone [28]. SST-2 is the GAP for GPA-1 and it is the most important gene product for pheromone desensitization and re-entry into the cell cycle [28,29]. We applied this paradigm of GPCR-ligand induced RGS gene expression to identify and characterize G protein signaling in liver during fasting and refeeding because we had made several observations indicating that Gq and RGS proteins influenced and responded to changes in liver metabolism.

We found that knockout mice deficient in either Gαq or its close paralog, Gα11 [30], exhibited abnormalities in liver regeneration following partial hepatectomy (Yu and Wilkie, unpublished observation). Given that RGS proteins are essential regulators of Ca^+2 ^signaling evoked by Gq/11-coupled agonists [19,21,31], we reasoned that a RGS gene might be induced in response to activation of Gq/11 signaling during liver regeneration. We found Rgs16 was rapidly up regulated following partial hepatectomy, suggesting a role in the metabolic response to the sudden loss of 70% of the liver. Therefore, we screened the livers of fasted and refed mice for differential regulation of RGS genes. Interestingly, of the 20 cloned RGS genes, only Rgs16 mRNA was induced in liver during fasting. Our studies described herein demonstrate that Rgs16 is a diurnally regulated gene in periportal hepatocytes of the liver, its expression is regulated by feeding and dietary constituents, and Rgs16 can be used as a biomarker to investigate G-protein pathways in liver regulating energy homeostasis.

## Results

### Diurnal regulation of Rgs16 mRNA in liver

**Figure 1:**
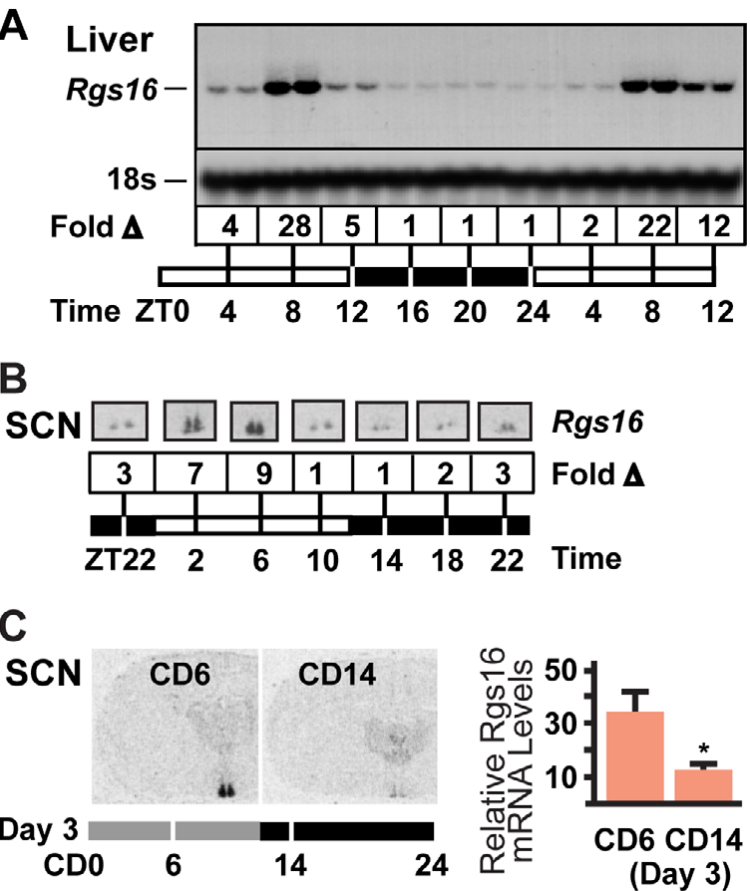
**Diurnal regulation of Rgs16 mRNA in liver and SCN**. ***(A) ***Northern blot analysis of liver total RNA (20 μg/lane) isolated from individual female mice sacrificed at the indicated times (ZT, Zeitgeber Time; 12 hr light phase, ZT0-12; 12 hr dark phase, ZT12-24). Two mice collected at each time point were pair-caged, food and water *ad libitum *(representative of >8 mice/time point). Fold change (Δ) in Rgs16 mRNA levels are relative to basal expression at ZT16 (assayed by densitometry). To confirm equal loading in all lanes, the filter was rehybridized with a radiolabeled oligonucleotide complementary to 18s rRNA. ***(B) ****In situ *hybridization of Rgs16 mRNA expression in SCN of *ad libitum *fed mice, 12 hr L:D. Fold change (Δ) in Rgs16 mRNA levels are relative to basal expression at ZT10 and ZT14. Relative Rgs16 mRNA levels were determined by densitometry of multiple sections obtained from each of two mice assayed at each time point. ***(C; left) ***Rgs16 mRNA expression (assayed by isotopic *in situ *hybridization) in coronal hemisections of SCN. *Ad libitum *fed mice were housed in constant darkness (CD) for two days prior to assay on day 3 at 6 hr into the presumptive light phase (CD6) and 2 hr into the presumptive dark phase (CD14). ***(C; right) ***Relative Rgs16 mRNA levels in SCN determined by densitometry of 2 sections from each of 4 mice per time point. Expression levels at CD6 and CD14 in Fig. 1C are similar to ZT6 and ZT14 in Fig. 1B, as expected of light entrained gene expression in SCN.

The diurnal expression of Rgs16 was characterized in liver of *C57BL/6 *female mice (pair caged) with free access to food and water under conditions of 12 hr light/dark, and sacrificed every 4 hrs at nine time points throughout a 36-hour period (Fig 1A). Interestingly, Rgs16 mRNA accumulated toward the end of the light phase, just prior to when animals begin feeding. Rgs16 mRNA returned to basal levels during the dark phase, presumably after feeding, and remained at basal levels until the middle of the next light phase. Rgs16 mRNA levels were always up regulated by eight hr into the light phase (Zeitgeber time 8 hr; ZT8) but some variation in expression was observed at ZT12 (Fig 1A, compare ZT12 Day 1 *vs *Day 2). About half of the *ad libitum *fed mice we assayed at ZT12 expressed high levels (similar to ZT8) of Rgs16 mRNA in liver (n > 40), whereas the mice with lower expression probably began feeding prior to lights out (see below). The diurnal rhythm in Rgs16 expression was observed in male and female mice and rats ranging in age from four weeks to more than one year. Diurnal oscillation in mRNA expression in liver was not observed for any of the other 19 RGS genes expressed in mice [32].

Rgs16 is one of a small subset of genes that is diurnally regulated in both liver and the hypothalamic suprachiasmatic nuclei (SCN), site of the central circadian pacemaker [33,34]. We showed by *in situ *hybridization that Rgs16 mRNA is expressed in the SCN of *C57BL/6 *female mice in our colony in the same temporal pattern as previously reported (Fig. 1B; [35]). Furthermore, this temporal pattern of Rgs16 expression in the SCN is maintained in *ad libitum *fed mice housed in constant darkness (CD) for two days prior to sacrifice on the third day; expression is elevated at 6 hr into the presumptive light phase (CD6) and declines to basal levels by 2 hr into the presumptive dark phase (CD14; Fig. 1C). Thus, Rgs16 expression in the SCN appears to be entrained similarly to other circadian regulated genes [35,36].

### Regulation of Rgs16 by fasting and refeeding

**Figure 2:**
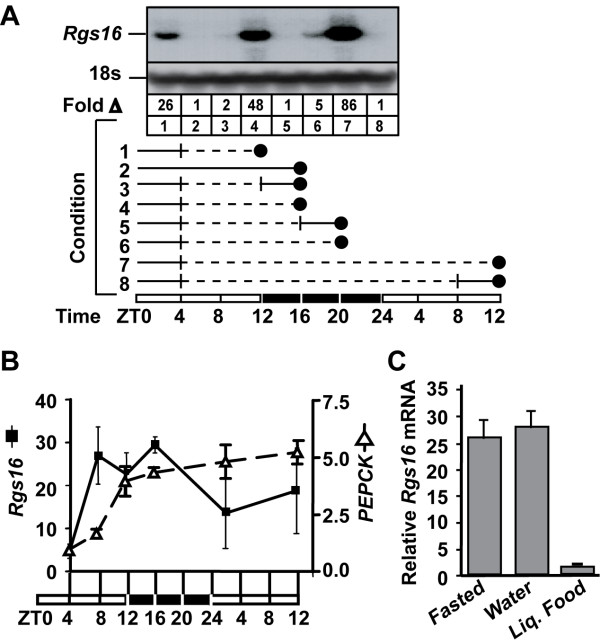
**Rgs16 mRNA regulated in liver by fasting and refeeding**. **(*A*) **Northern blot analysis of liver total RNA (20 μg/lane) isolated from individually caged female mice (*C57BL/6*) sacrificed at the indicated times (Representative of ≥10 mice/condition). Rgs16 accumulates in fasted mice in either light (lanes 1 and 7) or dark phase (lane 4); Rgs16 is basal within 4 hr of feeding in dark (lanes 2,3,5) or light phase (lane 8). Rgs16 mRNA declines in fasted mice near the end of dark phase (lane 6) before rebounding in the next light phase with continued fasting (lane 7). Solid line represents food and water available *ad libitum*; dashed line, food removed (water available *ad libitum*); vertical bar, time when food was removed or provided; solid circle, time of sacrifice. Time line: 12 hr light (open bar), 12 hr dark (filled bar). To confirm equal loading in all lanes, the filter was rehybridized with a radiolabeled oligonucleotide complementary to 18s rRNA. **(*B*) **QPCR of Rgs16 and *PEPCK *mRNA from liver of fasted mice. Food was removed at ZT4, and liver samples were collected at the indicated times (n = 6/time point, 3 females/cage) or **(*C*) **QPCR of Rgs16 mRNA of mice fasted from ZT4 and then fed by gavage (1 ml) at ZT14, either with water or complete liquid diet; mice sacrificed at ZT16; n ≥ 6, 3 females/cage. Fold induction compared to pooled RNA from fed mice (as in Fig. 2A, condition 2). ZT, Zeitgeber Time.

To determine whether Rgs16 mRNA expression in liver was upregulated in anticipation of a meal, but returned to basal levels after feeding, we examined the effect of withholding food on Rgs16 mRNA levels and refeeding at different times in the light cycle. Fasting mice for various intervals caused an increase in Rgs16 mRNA expression through the early-middle hours of the first dark phase (Fig. 2A, lanes 1,4). The induction of Rgs16 mRNA expression early in the fasting period was consistently observed in separate groups of female mice fasted from ZT4; but expression levels began to vary as the mice were fasted through the dark phase until ZT12 of the following day (Fig. 2A, lanes 6–7; Fig. 2B; solid line). By contrast, phosphoenolpyruvate carboxykinase (*PEPCK*) expression never declined at any time in any of those fasted mice (Fig. 2B; dashed line). In all cases, Rgs16 (and *PEPCK*) mRNA expression returned to basal levels within four hours of providing chow *ad libitum *(Fig 2A, conditions 3,5,8) or within 90 min after gavage of a complete liquid diet (Fig. 2C). Gavage with water did not affect Rgs16 expression, suggesting that stomach dilation or gastrointestinal motility are not factors regulating liver Rgs16 expression during fasting and refeeding.

### Rgs16 mRNA in periportal hepatocytes

**Figure 3:**
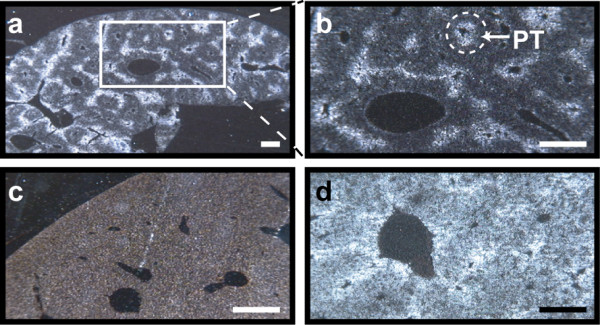
**Rgs16 mRNA is expressed in periportal hepatocytes**. *In situ *hybridization (dark field) of (*A-C*) Rgs16 and (*D*) *PEPCK *mRNA expression in liver. **(*A-B*) **Rgs16 mRNA is expressed specifically in periportal hepatocytes of mice fasted from ZT4 and sacrificed at ZT16 (12 hr L:D). **(*C*) **Rgs16 was not expressed in liver of fed mice. **(*D*) ***PEPCK *mRNA expression was observed in hepatocytes throughout the liver of fasted mice (ZT4-ZT16; same liver as *3A*, neighbouring section), although most abundantly in periportal hepatocytes. ZT, Zeitgeber Time. Scale bar = 100 μm.

Metabolic functions of the liver are distributed between zones of hepatocytes organized within acini 37]. Lypolysis and gluconeogenesis occur preferentially in periportal hepatocytes, whereas glycolysis and lipogenesis occur preferentially in hepatocytes surrounding the central vein 37]. The hepatic cell types that expressed Rgs16 mRNA were identified by *in situ *hybridization. Interestingly, Rgs16 expression in fasted mice was localized to periportal hepatocytes that surround the portal triad, consisting of a portal vein, a hepatic artery, and a bile duct (Fig. 3A,B; same condition as Fig 2 condition 4). Rgs16 expression remained tightly restricted to periportal hepatocytes even after a 24 hr fast. By contrast, *PEPCK *mRNA was expressed in hepatocytes throughout the liver of fasted mice, although it was most abundant in periportal hepatocytes (Fig 3D). Periportal expression of Rgs16 and *PEPCK *was confirmed by histological examination. Consistent with previous Northern analyses, Rgs16 mRNA expression was not detected by *in situ *hybridization in liver of fed mice (Fig 3C; same conditions as Fig 2, lane 2). In additional control experiments, sense probes of Rgs16 and *PEPCK *did not hybridize to adjacent sections (data not shown). The restricted pattern of Rgs16 expression to periportal hepatocytes suggests that Rgs16 may regulate lipolysis and/or gluconeogensis in liver.

### Restricted feeding shifts circadian expression of Rgs16 in liver but not SCN

**Figure 4:**
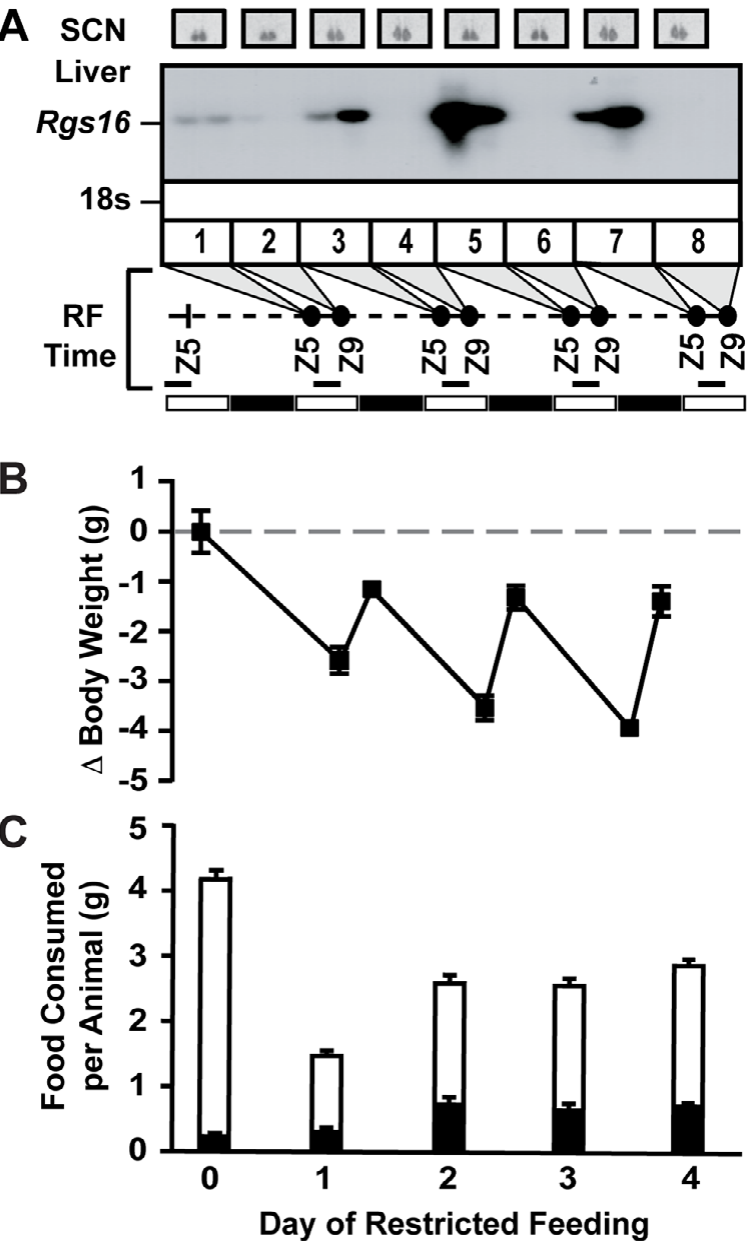
**Restricted feeding (RF) effects on Rgs16 mRNA expression, body weight, and feeding**. ***Top: ***Rgs16 mRNA expression in SCN (*in situ*) and liver (Northern, 20 μg/lane) of fasted (Z5) and fed (Z9) pair-caged female C57BL/6 mice. Zeitgeber time line, 12 hr light (open bar), 12 hr dark (filled bar). Solid line, normal chow *ad libitum *fed; dotted line, fasted; RF conditions, *ad libitum *fed only during Z5–Z9. ***Middle: ***changes in body weight before and after feeding during RF (avg 22 g at start of experiment, n = 46). ***Bottom: ***Day 0 is *ad libitum *fed mice, open bar indicates total food consumed per mouse per day; black bar indicates food consumed in first 30 min of the dark phase (7% of total), n = 12 pair-caged mice over 4 days. Day 1–4 (RF), open bar indicates total food consumed in 4 hr, Z5–Z9; black bar is food consumed in first 30 min (day 1, 31% of total; day 2-4, 38% of total; n = 46). Z, Zeitgeber time; RF, restricted feeding

Regulation of Rgs16 by fasting and refeeding suggested that restricted feeding (RF), during 4 hours of the light phase (ZT5-ZT9), might shift Rgs16 expression in liver along with transcription factors that control the circadian clock in peripheral tissues [35]. We characterized Rgs16 mRNA expression in *C57BL/6 *female mice over the first 4 days of RF (Fig. 4). As expected, Rgs16 was expressed in liver of fasted mice and declined to basal levels by the end of the 4 hr feeding period (ZT5-ZT9). Restricted feeding advances and intensifies Rgs16 expression in liver by the second or third day of RF (Fig. 4), coincident with increased locomoter activity as mice learn to anticipate feeding during the light phase [38].

On the first day of RF, Rgs16 mRNA expression in liver is no higher at ZT5 than in *ad libitum *fed mice (see Fig. 1, lane 1, 2), even though the RF mice were fasted through the dark phase and had lost 2.5 gm from their starting weight (Fig. 4, middle panel). This may reflect an underlying circadian regulation of liver gene expression as clock genes show a similar response on the first day of RF [35,36,38]. During the first 4 hr feeding period (day 1), female mice ate about 1.5 gm of chow and regained about 1 gm body weight (Fig. 4, bottom panel). However, they ate about half as much on day 1 as on subsequent days, presumably because they had not yet learned to anticipate fasting during the dark phase and feeding during the light phase. By day 2 RF, prior to feeding at ZT5, mice lost about 3.5 gm of their original weight, but they were alert and active, and ate voraciously upon refeeding, consuming about 0.7 gm chow within the first 30 min, 2.5 gm during 4 hr. This behavior was repeated days 3 and 4 of RF, and each day mice regained body weight to within about 1 gm of their starting weight.

Oscillations in Rgs16 mRNA expression are slightly delayed in the liver relative to the SCN in *ad libitum *fed mice maintained in a 12 hr light-dark cycle [35,36,38]. By contrast, restricted feeding during the light phase advances and intensifies Rgs16 expression in liver but does not alter expression in SCN (Fig. 4, top panel) presumably reflecting separate inputs of nutrients and light regulating Rgs16 expression in liver and SCN.

### Transcriptional control of Rgs16 gene expression

**Figure 5:**
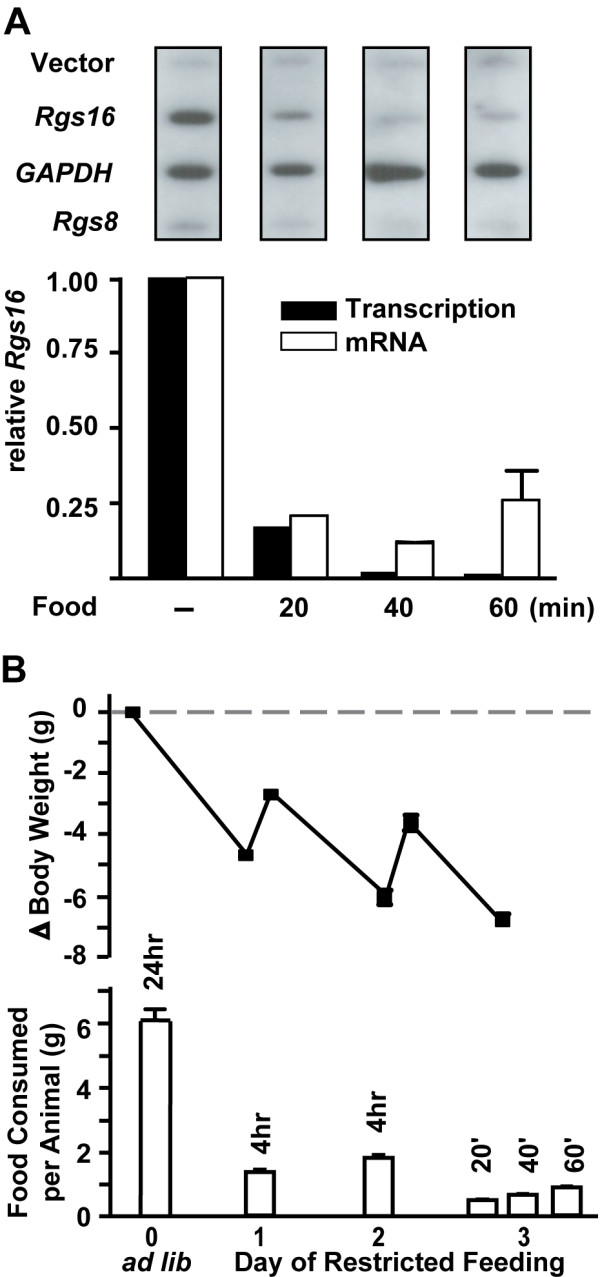
**Rgs16 gene transcription regulated by fasting and refeeding**. **(*A*) **Liver nuclei were prepared from fasted male *C57BL/6 *mice (n = 4/group; individually caged) at ZT5 on day 3 of RF, or refed for 20, 40, or 60 minutes. Liver nuclei from each condition were pooled prior to nuclear run-on. Nascent transcripts of radiolabeled RNA from nuclear run-ons were hybridized to plasmid cDNAs (5 μg) of mouse Rgs16, rat *GAPDH*, mouse *Rgs8 *or vector control. The relative transcriptional activity and QPCR assay of mRNA levels of Rgs16 in livers of fasted and refed mice is shown below the corresponding nuclear run-on blot. The results are representative of 3 separate experiments. **(*B*) **Changes in body weight and food intake during *ad libitum *(*ad lib*) and RF feeding in the male mice assayed in part A. Day 0, open bar indicates the average daily food consumption of *ad libitum *fed male mice (avg 27 g at start of experiment, n = 31 individually caged mice assayed over 4 days prior to RF); Day 1–2 (RF), open bar indicates total food consumed in 4 hr, ZT5–ZT9 (n = 31); Day 3 (RF), open bars indicate the amount of food consumed *ad libitum*, starting at ZT5 and feeding for 20, 40 or 60 min (n = 4/time point). ZT, Zeitgeber Time; RF, restricted feeding.

Transcription of the Rgs16 gene is induced during fasting and is inhibited after feeding. Body weight and food consumption of male *C57BL/6 *mice (individually caged) under RF conditions is shown (Fig. 5B). On day 3 of RF, fasted males were sacrificed at ZT5, or allowed to feed for 20, 40, or 60 min before sacrifice. Nuclear run-on assays showed that Rgs16 gene transcription declined about 5-fold from fasted levels within 20 min, and was not detectable above background by 60 min (Fig. 5A). By contrast, transcription of GAPDH, Rgs8, cyclophilin, and Gα11 remained essentially constant in liver of fed and fasted mice (Fig. 5A, and data not shown). Rgs16 mRNA levels declined about 4-fold within the first 20 min after refeeding, consistent with the decline in transcription during the same interval.

### Rgs16 mRNA and protein are rapidly down regulated after feeding

**Figure 6:**
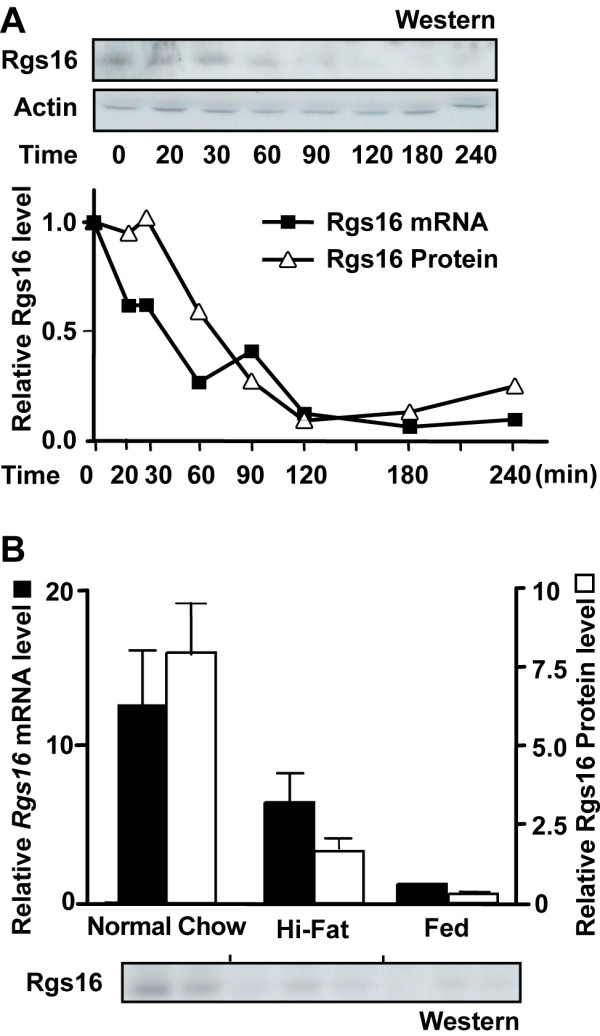
**Rapid postprandial decline of Rgs16 mRNA and protein in liver**. **(*A*) **Time course of Rgs16 mRNA and protein degradation in liver upon feeding at the beginning of the dark phase (ZT12). Food was removed from individually caged mice (female *C57BL/6*) at ZT4. Mice were fasted (time = 0) or refed for the indicated time (minutes), starting from ZT12. After sacrifice, liver samples were collected for mRNA and protein assays. Total liver protein and RNA was prepared from pooled samples of equal amount within each time point for Western blot and QPCR analysis, respectively (n = 3 mice/group). All mice had free access to water; 12 hr L:D. **(*B*) **Rgs16 mRNA and protein in liver of fasted mice is induced to different levels in mice maintained on normal chow or a high fat diet. Mice were maintained on high fat diet (Hi-Fat) for 7 weeks. Mice were fasted from ZT4 to ZT16 or *ad libitum *fed with normal chow from ZT12. Liver was collected at ZT16. Rgs16 protein expression was assayed by Western blot (bottom) and QPCR. Rgs16 mRNA and protein were individually assayed and quantitated by QPCR (*filled bar*) and Western blot densitometry (*open bar*; n = 3 mice/group) relative to pooled samples from fed mice. All mice (female *C57BL/6*) had free access to water; 12 hr L:D. ZT, Zeitgeber Time.

Rgs16 mRNA and protein expression in liver after a short fast was somewhat higher in mice maintained on normal chow compared with a high fat diet (Fig. 6). Feeding down regulated both Rgs16 mRNA and protein to basal levels within four hours (Figs. 2A, 6A, and data not shown). To compare the decline in Rgs16 mRNA and protein levels after feeding, female mice were fasted from ZT4 to ZT12; then, provided chow *ad libitum *at ZT12, and sacrificed to collect liver at the indicated times (Fig. 6A). Rgs16 mRNA and protein levels declined concomitantly within 20 min after feeding, falling to nearly basal levels within 60 min. A rapid decline in Rgs16 mRNA expression was also observed when female mice were refed by gavage (1 ml) of a complete liquid diet at ZT12 (Fig. 1). In summary, refeeding a complete chow or liquid diet down regulates Rgs16 in male and female mice (n >100) and rats (n = 6).

## Discussion

Rgs16 is one of the few genes whose expression oscillates in a circadian pattern in both liver and SCN (Figs. 1, 2, 3, 4) [35]. Rgs16 and other oscillatory genes in liver are synchronized by feeding time, whereas SCN neurons presumably respond to light stimulated neurotransmitter release at synaptic junctions with neurons from the retinal-hypothalamic tract. Many of the genes that oscillate both in liver and SCN are transcription factors integral to the circadian clock [35,36,38,39], whereas Rgs16 is a GAP for G alpha proteins of the Gi and Gq class [31,40,41]. Rgs16 and closely related RGS proteins in the R4 family negatively regulate G protein signaling [18], and can feedback inhibit Gi/Gq signaling by uncoupling hormone binding from effector protein activation in a variety of primary cell types [19,21,31]. The expression pattern and enzymatic activity of Rgs16 identifies it as a candidate regulator of Gi/Gq signaling during the transitions from fasting to feeding in liver and between light and dark phases in SCN.

G protein-coupled ligands stimulate expression of RGS genes in eukaryotes from mammals to yeast [18,22-24]. For example, the yeast mating pheromones are GPCR-ligands that induce both cell cycle arrest and the transcription/translation of the yeast RGS protein *SST-2*, which is required for rapid desensitization to mating pheromone and re-entry to the cell cycle [28]. Because the early phase of liver regeneration is impaired in mutant mice deficient in Gq signaling (data not shown), we applied the yeast paradigm as our rationale for screening the expression of all RGS genes in liver of fed and fasted mice to identify Gi and/or Gq signaling pathways regulating liver metabolism.

We found that Rgs16 is the only RGS gene (of 20 total [17]) that is diurnally expressed in liver of *ad libitum *fed mice (Fig. 1). Mice are nocturnal, and when provided *ad libitum *access to food and water, they eat nearly 80% of their daily food during the dark phase in a 12 hr light:dark (12hL:D) cycle. Rgs16 mRNA accumulates in liver in anticipation of a meal, either at the end of the light phase in mice maintained on a 12 hr L:D cycle (Figs. 1, 2), or prior to feeding at ZT5 on day 2 and subsequent days of restricted feeding (RF; Fig. 4). Furthermore, the amplitude of daily oscillations in Rgs16 mRNA and protein expression are modulated by energy deficiency, either increased in fasted mice whose body weight is significantly below set point (Fig. 4) or decreased in over weight mice maintained on a high fat diet (Fig. 6A). Importantly, the rate of Rgs16 gene transcription is induced by fasting and declines to basal levels shortly after feeding (Fig. 5). Coincident with these changes in transcription, Rgs16 mRNA and protein levels decline within 20 minutes after feeding begins, and drop to basal levels within 120 minutes (Fig. 6). Interestingly, if mice are not allowed to continue eating, but are restricted to the amount they can consume within 30 minutes of refeeding, Rgs16 mRNA expression returns within 120 min (data not shown). The tight localization of expression to Zone 1 periportal hepatocytes (Fig. 3) places Rgs16 at an ideal location to be regulated by orexigenic and satiety signals from the gut, the peripheral and/or central nervous system. The dynamic and localized expression of Rgs16 could be regulated by hormones and/or metabolites controlling liver metabolism.

The liver helps maintain whole body energy homeostasis in part by metabolism of glucose and fatty acids. Catabolic and anabolic metabolism in liver is regulated in response to daily repetitions of fasting and feeding, and the availability of energy stores in liver, adipose, and muscle. Glucagon and insulin are classical hormone regulators of the fasted and fed states 42, 43]. During fasting, glucagon simultaneously stimulates gluconeogenesis while inhibiting fatty acid synthesis in liver [44]. Glucagon signaling in hepatocytes is transduced by Gαs, which stimulates adenylyl cyclase to produce the second messenger cAMP, thereby activating the cAMP responsive transcription factor CREB [45,46]. However, Rgs16 is not a Gs-GAP and does not directly regulate Gs activity [18]; rather, Rgs16 may help integrate Gi- and/or Gq-signaling with glucagon or insulin signaling in liver.

Guided by the paradigm of the yeast mating pathway, we hypothesize that Rgs16 expression is induced in liver during fasting by activation of the Gi/Gq pathway(s) that Rgs16 protein negatively regulates. We propose that a preprandial agonist activates a hypothetical GPCR pathway that induces Rgs16 gene transcription and, possibly, promotes Rgs16 mRNA stability. Curiously, Rgs16 protein is not abundantly expressed in liver of fasted mice. We note that Rgs16 and other RGS proteins of the R4 subfamily typically are weakly expressed in many cell types and tissues despite abundant mRNA expression [47]. One possible explanation for this pattern is Rgs16 mRNA accumulates during fasting and is poised for translation, dependent on other dietary factors.

Rgs16 is specifically expressed in periportal hepatocytes, the oxygen-rich zone of the liver where lipolysis and gluconeogenesis predominates, suggests Rgs16 may regulate Gi/Gq pathway(s) that stimulate fatty acid oxidation and/or glucose production in liver. Because refeeding rapidly terminates Rgs16 gene transcription and allows Rgs16 mRNA and protein degradation, Rgs16 might inhibit Gi/Gq signaling during the transition from the fasted to fed states. In this model, Rgs16 functions as a switch, turning off preprandial signaling in liver once feeding has commenced. Given that the hepatic expression of Rgs16 and circadian clock genes rapidly and coordinately adjust to changes in feeding schedules, Rgs16 might regulate the feeding cues that reset the circadian oscillator in liver.

## Conclusion

Rgs16 is the only RGS gene (of 20 total) that is diurnally expressed in liver of *ad libitum *fed mice. Restricting feeding entrained Rgs16 transcription and mRNA expression in liver but did not affect circadian regulation of Rgs16 expression in the SCN. Thus, separate inputs regulate Rgs16 expression in liver and SCN. Rgs16 gene transcription, mRNA and protein levels are rapidly down regulated by feeding. The expression pattern and enzymatic activity of Rgs16 identifies it as a candidate regulator of Gi/Gq signaling during the transitions from fasting to feeding in liver and between light and dark phases in SCN.

## Materials and methods

### Materials

[^32^P]-dCTP was purchased from Amersham Bioscience (Piscataway, NJ). TRIZOL was purchased from Invitrogen (Carlsbad, California) and GeneScreen nylon membranes were obtained from New England Nuclear (Shelton, CT). All other lab supplies were purchased from Sigma (St Louis, MO) or Fisher (Hampton, NH).

### Animal and colony conditions

Mice were maintained at 20°C under a standard 12-hour light:dark cycle (12h L:D, lights on at 5 am). *C57BL/6 *mice were purchased from Jackson Laboratories (Bar Harbor, Maine). Female *C57BL/6 *mice in feeding studies were pair-caged unless otherwise indicated; male mice were caged individually during restricted feeding studies. Mice were *ad libitum *fed standard mouse chow (Normal Chow) containing 6% total energy as fat (Teklad 7002, Harlan Teklad Laboratories, Indianapolis, IN), except where noted. The high-fat diet contains 41% calories as fat-40% carbohydrate-19% protein (Teklad #96001). Mice were six to eight weeks of age before being switched from a normal chow to high fat diet. Experimental research on animals followed internationally recognized guidelines and had UT Southwestern IACUC approval (APN#0602-05-01-2).

### Real-time quantitative PCR

mRNA expression levels were determined using real time quantitative polymerase chain reaction. Cyclophilin was used as the normalizing gene. QPCR primers were designed using the Primer Express Software v 2.0 (Applied Biosystems, Foster City, CA) from published mRNA sequences. Rgs16 (NM_011267) QPCR primers: Forward – 5'cctggtacttgctactcgctttt3'; Reverse – 5'agcacgtcgtggagaggat3'; Cyclophilin (M60456) QPCR primers: Forward – 5'tggagagcaccaagacagaca3'; Reverse – 5'tgccggagtcgacaatgat3'. Total RNA was extracted from 50 mg liver as described below in Northern Blot Hybridization. Following DNase treatment, cDNA was synthesized from total RNA (2 μg) in 100 μL reactions using Superscript II reverse transcriptase kit (Invitrogen, Carlsbad, CA) and stored at -20°C until use. Polymerase chain reaction amplifications were performed in 96-well optical reaction plates (ABI) on the ABI Prism 7000 Sequence Detection system using the SYBR-Green (ABI) reaction conditions (1 cycle at 50°C for 2 min, 1 cycle at 95°C for 10 min, 40 cycles at 95°C for 15 sec and 60°C for 1 min), the baseline and threshold were set to experimentally determined values and the data were analyzed using the Comparative C_T _method (2−ΔΔCT
 MathType@MTEF@5@5@+=feaafiart1ev1aaatCvAUfKttLearuWrP9MDH5MBPbIqV92AaeXatLxBI9gBaebbnrfifHhDYfgasaacH8akY=wiFfYdH8Gipec8Eeeu0xXdbba9frFj0=OqFfea0dXdd9vqai=hGuQ8kuc9pgc9s8qqaq=dirpe0xb9q8qiLsFr0=vr0=vr0dc8meaabaqaciaacaGaaeqabaqabeGadaaakeaacqaIYaGmdaahaaWcbeqaaiabgkHiTiabfs5aejabfs5aejabboeadnaaBaaameaacqqGubavaeqaaaaaaaa@33ED@) as described [32]. Relative Rgs16 mRNA fold induction was calculated compared to pooled RNA from fed mice (as in Fig. 2A, condition 2).

### Northern blot hybridization

At time of collection, mice were sacrificed by cervical dislocation and individual livers were dissected, frozen in liquid nitrogen immediately, and stored at -80°C for future analysis. RNA extraction was performed using TRIZOL according to manufacture's protocol and liver RNA (20 μg per lane) was used for Northern analysis. Briefly, RNA samples were size separated by electrophoresis on a 1.0% agarose denaturing gel, transferred to a nylon membrane overnight, and probed with Rgs16 cDNA in 50% formamide. Radionucleotide hybridization probes were either random primed (Rgs16 cDNA; complete open reading frame) or end-labeled (18s rRNA oligonucleotide [GCCGTGCGTACTTAGACATGCATG]) as described [48]. Northern blot hybridization filters were prehybridized at 42°C for six hours, hybridized overnight at 42°C, and then the membrane was washed twice in 2X SSC at 25°C, once in 2X SSC/2% SDS at 55°C, and once in 0.1X SSC at 25°C; filters were dried and exposed to Fuji RA film for at least 16 hours. Fold change (Δ) in Rgs16 mRNA levels are relative to basal expression at D12 (assayed by densitometry).

### In situ hybridization

Mice were sacrificed by cervical dislocation and the livers were rapidly frozen on dry ice and stored at -80°C. Fresh frozen sections were cut in a cryostat and thaw-mounted onto SuperFrost Plus glass slides (Fisher Scientific, Pittsburgh, PA). Sections were pre-treated as described, including fixation, acetic anhydride and defatting steps [49]. Slides with cover slip were incubated for 18 hrs at 60°C in a humidified chamber with buffer containing denatured salmon sperm DNA (0.033 mg/mL), yeast tRNA (0.15 mg/mL), dithiothreitol (40 μM), and a cRNA at 1 × 10^7 ^cpm/mL, other conditions as described [49]. Riboprobes were generated using an in vitro transcription kit (Ambion; Austin, TX) by using T3 or T7 RNA polymerase in the presence of ^32^P-UTP, and purified using RNA quickspin columns. Sections were then treated with RNase A (20 mg/mL, 30 min at 45°C) and washed in descending concentrations of sodium citrate buffer to a stringency of 0.1X SSC at 60°C, and air-dried. Tissue sections were exposed to Biomax MR film (Kodak, Rochester, NY) followed by dipping in autoradiographic emulsion (NTB2, Kodak), exposed for an appropriate duration, developed, fixed, counterstained with cresyl violet acetate, a cover slip applied with DPX mounting media and visualized under bright and dark field microscopy (Olympus, Melville, NY). Adjacent sections from fresh-frozen livers were used as controls for the effects of perfusion on mRNA integrity and hybridization to sense probes.

### Western blots

Livers were rapidly excised and immediately frozen in liquid nitrogen. Liver (50 mg) was resuspended in 1% SDS that contained a cocktail of protease inhibitors, including leupeptin (10 μg/mL), soybean trypsin inhibitor (10 μg/mL), MG132 (16 μg/mL), phenylmethylsulfonyl fluoride (16 μg/mL), tosyl lysine choromethyl ketone (16 μg/mL), and tosyl phenylalanine choromethyl ketone (16 μg/mL). The samples were sonicated (Virtis, Gardiner, NY) and immediately boiled for three minutes. A Lowry protein assay was employed to quantitate the protein and ensure equal loading. The samples were separated by SDS-PAGE and transferred to nitrocellulose paper. Proteins were detected using Rgs16 antiserum (a gift from Carol Beadling, Cornell University). Rgs16 antiserum does not cross react with recombinant mouse Rgs4 or Rgs8 protein. Enhanced Chemiluminescence (Amersham-Pharmacia) was utilized to detect the rabbit HA-tagged secondary antibody. The membrane was exposed to ML film (Kodak Biomax) for 2–15 minutes. Autoradiographs were scanned with a BioRad Fluor-S MultiImager to determine signal intensities.

### Transcription run-on

Nuclei from mouse livers were isolated as described [50], resuspend in glycerol storage buffer (50 mM Tris-HCl pH 8.3, 5 mM MgCl_2_, 0.1 mM EDTA pH 8.0 and 40% glycerol) and flash-frozen in liquid nitrogen. Nuclei were stored at -80°C and used within 4 weeks. Mouse Rgs16, mouse Rgs8, rat GAPDH cDNA or empty plasmid vectors (5 μg) were slot-blotted onto nylon GeneScreen membrane (NEF 983, NEN life Science Products, Inc) according the manufacturer's protocol. The nuclear RNA elongation reaction, isolation of newly synthesized ^32^P-RNA, and hybridization to cDNA plasmids were performed as described 51]. The amount of hybridizing ^32^P-RNA was quantitated by densitometric scanning (using a Fujifilm FLA-5100 image reader) of phosphorimager screens exposed for 24 h. Data from different fasting and refeeding conditions were normalized to transcription of the GAPDH gene.

### Statistics

Experiments were conducted in groups of two or three mice per condition and repeated at least twice. Quantitative data for each condition or time point are represented as mean values ± SEM. GraphPad Prism software (GraphPad, San Diego, CA) was used to perform all statistical analyses. The two-tailed, unpaired Student's *t*-test was used to determine statistically significant differences (*P *< 0.05) between mean values.

## Competing interests

The author(s) declare that they have no competing interests.

## Authors' contributions

JH contributed to the QPCR analysis and did Western blots of fasting and refeeding experiments, and final formatting of all figures. VP carried out nuclear run-on and restricted feeding experiments. DMK did restricted feeding experiments. KY did the Northern blots and co-ordinated *in situ *hybridization. SJG did SCN *in situ *hybridization. TMW conceived of the study, participated in its design, execution, and coordination. All authors contributed figures, helped draft portions of the manuscript, and read and approved the final manuscript.
